# App-Controlled Treatment Monitoring and Support for Patients With Head and Neck Cancer Undergoing Radiotherapy: Results From a Prospective Randomized Controlled Trial

**DOI:** 10.2196/46189

**Published:** 2023-10-19

**Authors:** Tanja Sprave, Michelle Pfaffenlehner, Raluca Stoian, Eleni Christofi, Alexander Rühle, Daniela Zöller, Alexander Fabian, Harald Fahrner, Harald Binder, Henning Schäfer, Eleni Gkika, Anca-Ligia Grosu, Felix Heinemann, Nils Henrik Nicolay

**Affiliations:** 1 Department of Radiation Oncology University of Freiburg Medical Center Freiburg Germany; 2 German Cancer Consortium (DKTK), Partner Site Freiburg German Cancer Research Center (DKFZ) Heidelberg Germany; 3 Institute of Medical Biometry and Statistics Faculty of Medicine and Medical Center University of Freiburg Freiburg Germany; 4 Freiburg Center for Data Analysis and Modelling University of Freiburg Freiburg Germany; 5 Department of Radiation Oncology University of Leipzig Medical Center Leipzig Germany; 6 Comprehensive Cancer Center Central Germany, Partner Site Leipzig Leipzig Germany; 7 Department of Radiation Oncology University Hospital Schleswig-Holstein Kiel Germany

**Keywords:** mHealth, head and neck cancer, head and neck squamous cell carcinoma, HNSCC, radiotherapy, mobile app, quality of life, patient-reported outcome measure, PROM, mobile health, head, neck, cancer, oncology, radiation, randomized controlled trial, RCT, satisfaction, treatment surveillance, patient surveillance, feasibility, patient reported, outcome measure, app-based

## Abstract

**Background:**

Head and neck cancers (HNCs) are very common malignancies, and treatment often requires multimodal approaches, including radiotherapy and chemotherapy. Patients with HNC often display a high symptom burden, both due to the disease itself and the adverse effects of the multimodal therapy. Close telemonitoring of symptoms and quality of life during the course of treatment may help to identify those patients requiring early medical support.

**Objective:**

The App-Controlled Treatment Monitoring and Support for Patients With Head and Neck Cancer (APCOT) trial aimed to investigate the feasibility of integrating electronic patient-reported outcomes (ePROs) in the treatment surveillance pathway of patients with HNC during the course of their radiotherapy. Additionally, the influence of app-based ePRO monitoring on global and disease-specific quality of life and patient satisfaction with treatment was assessed.

**Methods:**

Patients undergoing radiotherapy for histologically proven HNCs at the Department of Radiation Oncology, University Medical Center Freiburg, Germany, were enrolled in this trial and monitored by weekly physician appointments. Patients were randomized between additional ePRO monitoring on each treatment day or standard-of-care monitoring. Feasibility of ePRO monitoring was defined as ≥80% of enrolled patients answering ≥80% of their daily app-based questions. Quality of life and patient satisfaction were assessed by the European Organisation for Research and Treatment of Cancer Core Quality of Life Questionnaire (QLQ-C30), the head and neck cancer module (H&N35), and the validated Patient Satisfaction Questionnaire Short Form (PSQ-18) at the completion of treatment and compared between trial arms.

**Results:**

A total of 100 patients were enrolled in this trial, and 93 patients were evaluable. All patients (100%) in the experimental arm answered ≥80% of the ePRO questions during treatment, reaching the predefined threshold for the feasibility of ePRO monitoring (*P*<.001 in the binomial test). No clinical or patient-specific factor was found to influence feasibility. Global health and most domains of the general quality of life were comparable between trial arms, but an increased HNC-specific symptom burden was reported by patients undergoing ePRO surveillance. ePRO monitoring resulted in improved patient satisfaction regarding interpersonal manners (*P*=.01), financial aspects (*P*=.01), and time spent with a doctor (*P*=.01).

**Conclusions:**

This trial demonstrated the feasibility of incorporating daily app-based ePRO surveillance for patients with HNC undergoing radiotherapy. Our data, for the first time, demonstrate that telemonitoring in this setting led to increased reporting of HNC-specific symptom burden and significantly improved several domains of patient satisfaction. Further analyses are needed to assess whether our findings hold true outside the context of a clinical trial.

**Trial Registration:**

German Clinical Trials Register DRKS00020491; https://drks.de/search/en/trial/DRKS00020491

## Introduction

Head and neck cancers (HNCs) are among the most common malignancies worldwide, affecting more than 740,000 patients and resulting in 360,000 deaths per year [1]. Treatment for nonmetastatic HNC depends on disease stage and localization and usually requires multimodal approaches comprising surgery, radiotherapy, and concomitant systemic treatments [2-4]. Within the treatment context, radiotherapy is a therapeutic mainstay, both as a primary treatment for patients with HNC and as an adjuvant therapy after surgery in case of locoregionally advanced cancers or increased risk of tumor recurrence [5-7]. Both the HNC itself and the required treatments often result in significant morbidity. Considering the improved oncologic outcomes, especially for distinct subgroups of patients with HNC, the resulting effects of cancer and its treatment on short-term and long-term quality of life become increasingly important after therapy [8,9].

Although cancer-related morbidity often requires upfront or even protective interventions prior to treatment initiation, such as a feeding tube or tracheostomy placement, treatment-related toxicities present with a delayed onset and may even occur with a significant delay after completion of therapy. Radiation-associated side effects are routinely monitored by the treating physicians several times during the course of radiotherapy and also regularly, at larger time intervals, during the follow-up period. Despite the considerable logistical efforts required for repeated physician appointments, there are several disadvantages to obtaining physician-reported outcome measures in patients with HNC. In the increasing gaps between assessments, cancer- or treatment-related symptoms may go unnoticed and patients may also become lost to follow-up. Additionally, patients may not adequately or completely report their symptoms during timed physician appointments [10,11].

It has been suggested that mobile apps may enable regular and close monitoring of patient-reported outcome measures (PROMs), such as disease symptoms and treatment-related toxicities, and in this respect, they may provide an additional tool to bridge the gap between physician appointments [12]. Several surveys have reported relatively high acceptance rates for mobile health apps among patients with cancer undergoing radiotherapy and providers of cancer care [13,14]. Additionally, 2 randomized clinical trials [15,16] have demonstrated improved overall survival through PROM telemonitoring in patients with advanced non–small cell lung cancers and metastatic tumor diseases. In both trials, close web-based symptom surveillance helped to detect symptoms indicative of cancer recurrence or deteriorating performance status, resulting in earlier initiation of salvage or supportive treatments. Similarly, in a randomized trial [17] investigating posttreatment care in lung cancer, patients followed up by electronic patient-reported outcome (ePRO) assessments experienced a significantly lower symptom burden and fewer posttherapeutic complications compared to patients receiving standard-of-care follow-up.

Considering both their high risk for locoregional recurrences and increased likelihood of experiencing higher-grade treatment-related toxicities, patients with HNC may derive particular benefits from an app-based collection of ePROs. In this respect, ePROs may provide a means to detect both recurrence symptoms and indicators of declining quality of life. The feasibility and potential benefits of app-based monitoring of patients with HNC have not yet been elucidated. Our App-Controlled Treatment Monitoring and Support for Patients With Head and Neck Cancer (APCOT) trial investigated, for the first time, the feasibility of daily app-based symptom monitoring in patients with HNC undergoing radiotherapy as well as patient compliance levels and the impact of ePRO monitoring on the global and disease-specific quality of life and patient satisfaction.

## Methods

### Ethical Considerations

This single-center prospective randomized controlled clinical trial was approved in advance by the Independent Ethics Committee of the University of Freiburg, Germany (reference number: 87/19), on November 26, 2019, and it is registered in the German Clinical Trials Register (DRKS00020491). It was carried out in accordance with the declaration of Helsinki in its current form.

### Patient Recruitment and Treatment

The inclusion criteria for this trial included histologically confirmed tumors in the head and neck region, scheduled radiotherapy or chemoradiation, as indicated by the respective multidisciplinary tumor board, an age ≥18 years, a Karnofsky performance score ≥50%, and provision of written informed consent. Patients with significant neurological or psychiatric diseases and patients unable to provide informed consent were excluded from this trial.

All patients with histologically confirmed cancers of the head and neck region, scheduled for (chemo)radiotherapy at the Department of Radiation Oncology, University of Freiburg Medical Center, Germany, were screened for inclusion in the trial. Consenting patients were randomized by block randomization between the standard-of-care monitoring and ePRO monitoring during the course of radiation treatment, and 50 patients were included in each trial arm. Protocol details have been published previously [18]. The trial was approved by the local data protection committee in advance, and all patient data were pseudonymized before storing and statistical analysis. Study participants did not receive any financial reimbursement for their participation in this trial.

### ePRO Monitoring During Radiotherapy

Patients in both trial arms received weekly physician appointments during the course of their outpatient treatment and additional appointments if they were medically indicated for the monitoring and treatment of symptoms occurring during radiotherapy. Patients in the experimental arm were additionally monitored by collecting daily PROs during outpatient treatment. General and disease-specific quality of life as well as treatment-related symptoms were investigated by a dedicated mobile app (myoncare, Oncare GmbH) on a departmental mobile device immediately prior to each radiotherapy fraction. PROM items were taken from the head and neck cancer module (H&N35) questionnaire developed and validated by the European Organisation for Research and Treatment of Cancer (EORTC) [19,20]. Patients were presented with 7 items from the questionnaire on each treatment day, and questions were rotated so that all items were answered weekly.

All patients in the experimental arm were asked about their need for a physician appointment on a daily basis through the app, and patients in the standard-of-care arm were offered daily physician appointments, if necessary, by the radiotherapist, as per institutional standards. No direct therapeutic measures were taken based on the collected ePROs, and all interventions were only indicated by the treating physician after a physical appointment in both treatment arms. Prior to inclusion in the experimental arm, all patients received an introductory session for the app by a study nurse.

### Assessment of Quality of Life and Patient Satisfaction

Global quality of life was assessed at the completion of radiotherapy using the paper-based EORTC QLQ-C30 questionnaire, and the quality of life and symptom burden for patients with HNC was quantified using H&N35. Patient satisfaction was measured upon completion of radiotherapy using the validated Patient Satisfaction Questionnaire Short Form (PSQ-18) on paper [21].

### Statistical Analysis

The feasibility of ePRO monitoring during radiotherapy was prespecified as the primary end point of this trial. As there is no uniformly accepted definition of feasibility for this type of trial, we have defined the feasibility threshold based on a previous trial from our group. Similar analyses have defined lower thresholds for feasibility, for example, 70% of patients answering 60% of questions, as reported by Tran et al [22]. As patients in our trial were monitored on all treatment days during treatment, we defined the feasibility threshold as the equivalent of answering all daily questions on 4 out of 5 treatment days each week.

Feasibility was assessed by the percentage of patients providing answers to ≥80% of ePRO items during the course of radiotherapy, and feasibility was defined as the ability of ≥80% of patients in the experimental arm to answer ≥80% of ePRO questions. Sample size calculation was based on an assumed percentage of 89% for the primary end point, and a sample size of 50 patients was calculated to provide a power of 80% (1-β) to a 1-sided significance level of 5% (α).

The primary end point was analyzed using a binomial test. Descriptive statistics were reported as means (SDs) in the case of continuous variables as well as absolute and relative numbers for categorical variables. Logistic regression models were used to estimate the effect of each covariate (eg, histology, smoking, systematic therapy, therapy, cardiovascular comorbidity, neurological comorbidity, nephrological comorbidity, diabetes, pulmonary comorbidity, and Charlson Comorbidity Index) on the odds of answering more than 80% of the questionnaires, including the experimental arm. The models were adjusted by age and gender.

Secondary end points for this trial were global quality of life and quality of life or symptom burden for patients with HNC as well as patient satisfaction at the completion of radiotherapy. Secondary end points were compared between both trial groups; for statistical analysis, sum scores for each functional and symptom scale were calculated from the questionnaires, as defined by the EORTC scoring manual and compared using Wilcoxon rank sum tests with continuity correction. *P* values <.05 determined statistically significant differences between groups.

The statistical analysis was conducted using R (version 4.2.2; R Core Team). For the effect size calculation, the R package effectsize (version 0.8.3) was used.

## Results

### Feasibility of App-Based Treatment Monitoring During Radiotherapy

**Figure 1:**
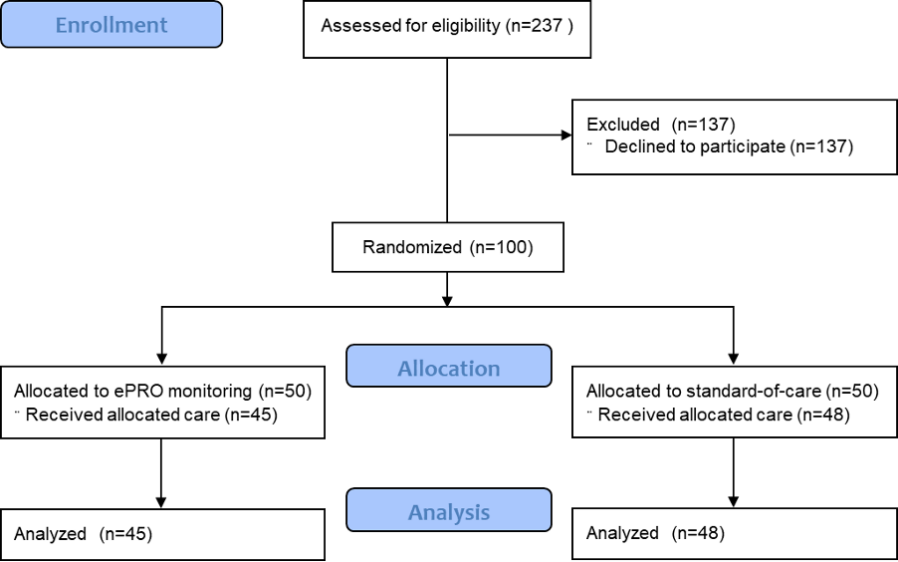
The CONSORT (Consolidated Standards of Reporting Trials) diagram for the App-Controlled Treatment Monitoring and Support for Patients With Head and Neck Cancer (APCOT) trial.

A total of 50 patients were enrolled in each arm of this trial, but 5 patients in the ePRO monitoring arm and 2 patients in the control arm had to be excluded from the final analysis due to withdrawal from the trial during the course of radiotherapy (Figure 1). The overall treatment time was comparable in the ePRO monitoring arm (mean 43.9, SD 9.55 days) and the standard-of-care arm (mean 43.49, SD 8.08 days). Patient characteristics of both trial arms can be found in Table 1. All patients in the experimental arm (100%) were able to answer ≥80% of ePRO questions during their treatment, reaching the predefined threshold for the feasibility of ePRO monitoring during radiotherapy (*P*<.001 in the binomial test). There was no reduction in answered questions during the course of treatment, and >80% of patients addressed ≥80% of ePRO items in each treatment week (*P*<.001). Therefore, the feasibility of app-based ePRO monitoring during radiotherapy in patients with HNC could be demonstrated in this trial.

**Table 1:** Patient and treatment characteristics (per-protocol patients).

Characteristics		ePRO^a^ monitoring (n=50)		Control (n=50)	
		Patients, n (%)	95% confidence limits	Patients, n (%)	95% confidence limits
Patients treated		45 (90)	0.579, 0.843	48 (96)	0.579, 0.837
Male gender		32 (71.1)	56.186, 63.594	34 (70.8)	64.178, 69.782
Age^b^		—^c^	0.157, 0.421	—	0.317, 0.599
Smokers		13 (28.9)	0.579, 0.843	22 (45.8)	0.579, 0.837
**Histology**					
	Nonkeratinizing squamous cell carcinoma	31 (72.1)	0.59, 0.852	32 (68.1)	0.549, 0.813
	Keratinizing squamous cell carcinoma	5 (11.6)	0.022, 0.21	10 (21.3)	0.097, 0.329
	adenocarcinoma	5 (11.6)	0.022, 0.21	2 (4.3)	–0.014, 0.1
	Other	2 (4.7)	–0.015, 0.109	3 (6.4)	–0.005, 0.133
**Tumor localization**					
	Oropharynx	15 (33.3)	0.195, 0.471	19 (39.6)	0.258, 0.534
	Oral cavity	13 (28.9)	0.157, 0.421	10 (20.8)	0.093, 0.323
	Larynx	6 (13.3)	0.034, 0.232	5 (10.4)	0.018, 0.19
	Hypopharynx	3 (6.7)	–0.006, 0.14	5 (10.4)	0.018, 0.19
	Nasopharynx	3 (6.7)	–0.006, 0.14	1 (2.1)	–0.02, 0.062
	Parotid glands	2 (4.4)	–0.016, 0.104	3 (6.2)	–0.006, 0.13
	Other	3 (6.6)	—	5 (10.4)	—
**Treatment**					
	Definitive radiotherapy	20 (44.4)	0.299, 0.589	21 (45.9)	0.298, 0.578
	Adjuvant radiotherapy	23 (51.1)	0.365, 0.657	22 (45.8)	0.317, 0.599
	Reirradiation	0 (0.0)	0, 0	1 (2.1)	–0.02, 0.062
	Palliative radiotherapy	2 (4.4)	–0.016, 0.104	1 (2.1)	–0.02, 0.062

^a^ePRO: electronic patient-reported outcome.

^b^The mean age for the electronic patient-reported outcome (ePRO) group was 60 (SD 12) years, and the mean age for the control group was 66 (SD 9) years.

^c^Not applicable.

### Factors Influencing Patient Compliance With the App-Based Treatment Monitoring

In a second step, clinical and patient-related covariates that could influence the feasibility of ePRO monitoring during radiotherapy in patients with HNC were analyzed. Patient age, gender, comorbidity burden (as assessed by the Charlson Comorbidity Index), the type of comorbidity (eg, cardiovascular, neurological, pulmonological, and nephrological), and smoking status did not show any correlation with the patient’s ability and willingness to undergo telemonitoring during radiotherapy. Similarly, tumor localization, tumor histology, or treatment concept did not influence the feasibility of ePRO monitoring. Detailed results can be found in Multimedia Appendix 1.

### Effects of ePRO Monitoring on the Quality of Life of Patients With HNC

**Figure 2:**
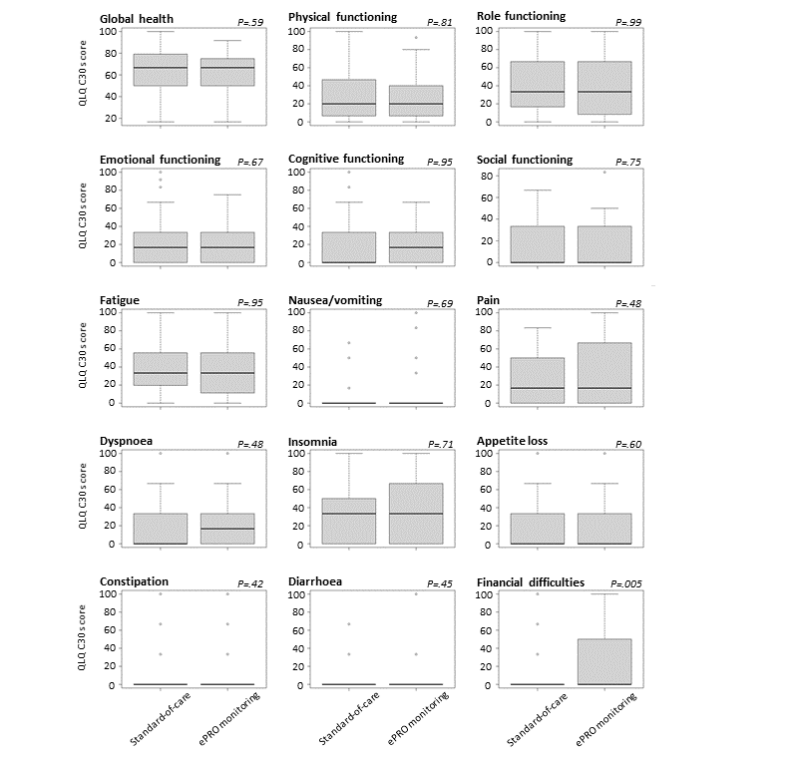
Comparison of functional and symptom scales upon completion of radiotherapy between patients undergoing electronic patient-reported outcome (ePRO) monitoring and those receiving standard-of-care monitoring. Items were assessed by the European Organization for Research and Treatment of Cancer Core Quality of Life Questionnaire (QLQ-C30) questionnaire. High values in the functioning scales represent a high level of functioning, whereas high levels in symptom scales represent a high level of symptom burden. Graphs depict median values (bold black lines) and IQRs.

Patient-reported quality-of-life items from the EORTC QLQ-C30 questionnaire and H&N35 at the time of radiotherapy completion were analyzed and compared between the trial arms. Global health was comparable between patients undergoing ePRO monitoring and those receiving standard-of-care monitoring (median 66.67 and *P*=.59 in Wilcoxon rank sum test with continuity correction). Similarly, there were no significant differences in most domains of quality of life between patients undergoing ePRO monitoring and those receiving standard-of-care monitoring (Figure 2). The only domain in which a difference between the trial arms was observed was that for financial difficulties; a higher level of financial difficulties was reported by patients in the ePRO arm (median 0 in both groups and *P*=.005). The effect size (ES) was based on the rank biserial correlation; it was *r*=–0.27, corresponding to a medium effect size.

**Figure 3:**
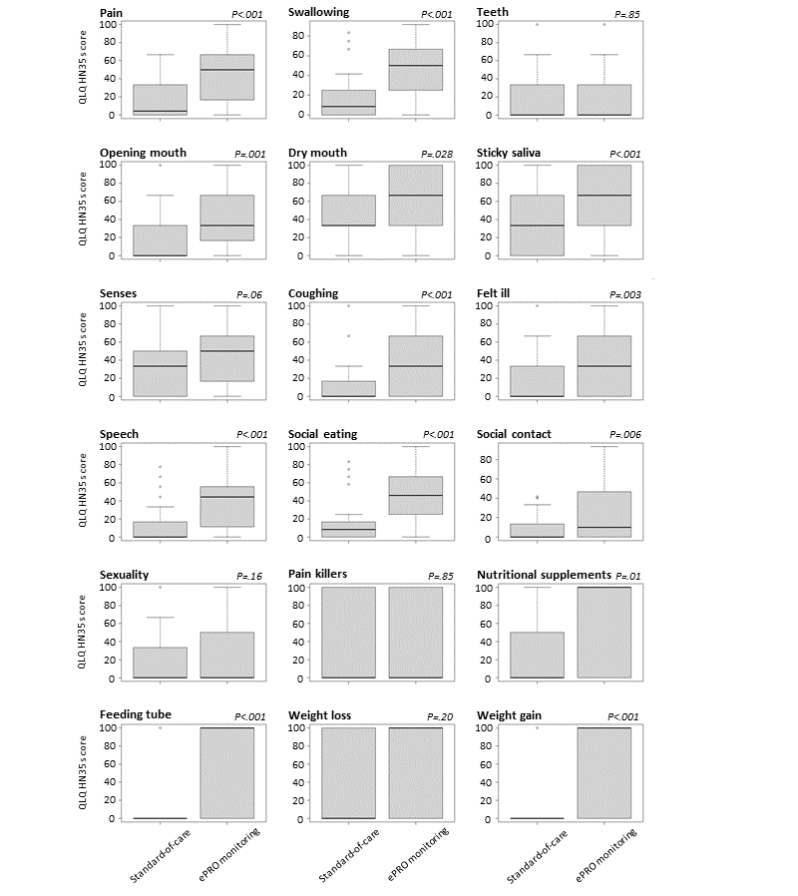
Comparison of symptom burden upon completion of radiotherapy between patients undergoing ePRO monitoring and those receiving standard-of-care monitoring. Items were assessed by the European Organization for Research and Treatment of Cancer Quality of Life Questionnaire Head and Neck Module (QLQ-H&N35) module. High values in the functioning scales represent a high level of functioning, whereas high levels in symptom scales represent a high level of symptom burden. Graphs depict median values (bold black lines) and IQRs.

In contrast, the HNC-specific symptom burden, as assessed by H&N35, appeared significantly higher in several domains for patients monitored daily using ePROs (Figure 3). Patients providing daily app-based data reported significantly increased pain (median 50), as compared to the control group (median 4.2; *P*<.001; *r*=–0.65; very large ES), swallowing impairments (median 50 vs 8.3; *P*<.001; *r*=–0.56; very large ES), dry mouth (median 66.7 vs 33.3; *P*=.03; *r*=–0.27; medium ES), and sticky saliva (median 66.7 vs 33.3; *P*<.001; *r*=–0.43; very large ES). Similarly, patients undergoing daily ePRO monitoring often indicated coughing (median 33.3 vs 0; *P*<.001; *r*=–0.42; very large ES), speech impairments (median 44.4 vs 0; *P*<.001; *r*=–0.49; very large ES), social eating (median 45.8 vs 8.3; *P*<.001; *r*=–0.64; very large ES), contacts (median 10 vs 0; *P*=.006; *r*=–0.34; large ES), and feeling ill (median 33.3 vs 0; *P*=.003; *r*=–0.36; large ES). Regarding nutrition during radiotherapy, patients in the ePRO arm reported an increased need for nutritional supplements (median 100 vs 0; *P*=.008; *r*=–0.29; medium ES), increased use of feeding tubes (median 100 vs 0; *P*<.001; *r*=–0.46; very large ES), and increased weight gain (median 100 vs 0; *P*<.001; *r*=–0.47; very large ES).

### Effects of the ePRO Monitoring on Patient Satisfaction in Patients With HNC

**Figure 4:**
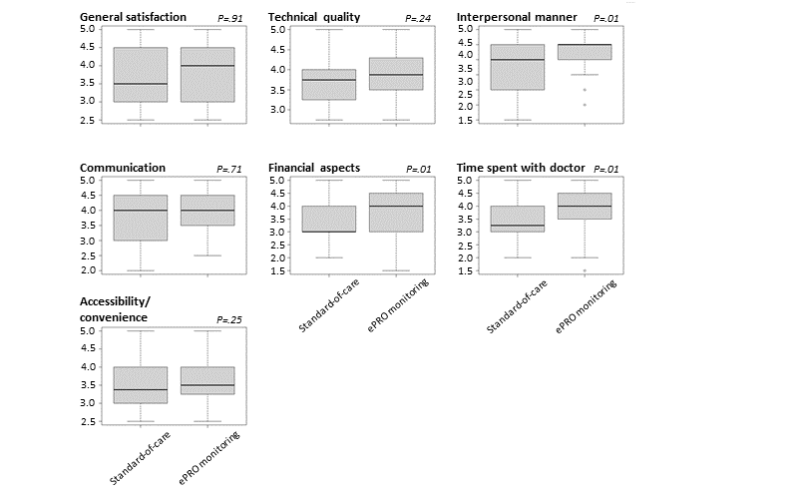
Comparison of domains of patient satisfaction upon completion of radiotherapy between patients undergoing electronic patient-reported outcome (ePRO) monitoring and those receiving standard-of-care monitoring. Items were assessed by the RAND Patient Satisfaction Questionnaire Short Form (PSQ-18) questionnaire. High values represent increased patient satisfaction. Graphs depict median values (bold black lines) and IQRs.

Considering the high compliance of patients with HNC undergoing ePRO surveillance during radiotherapy, patient satisfaction with treatment and medical care was assessed upon completion of radiotherapy using the PSQ-18 questionnaire. Patients undergoing daily ePRO monitoring reported increased satisfaction levels in 3 of the measured 7 domains (Figure 4). Although the general satisfaction levels were comparable between patients in the ePRO monitoring and standard-of-care arms (median 4 vs 3.5; *P*=.9), patients undergoing daily ePRO monitoring reported improved ratings regarding interpersonal manners (median 4.5 vs 4; *P*=.01; *r*=–0.31; large ES), financial aspects (median 4 vs 3; *P*=.01; *r*=–0.3; medium ES), and the time spent with a doctor (median 4 vs 3.25; *P*=.01; *r*=–0.31; large ES). The high satisfaction levels with medical care in the ePRO arm of the trial correspond well with the observed high compliance levels toward digital monitoring.

## Discussion

### Principal Findings

Our data demonstrate the feasibility of an app-based treatment monitoring and ePRO assessment during radiotherapy of HNC. Overall, high compliance rates could be demonstrated among patients, with >80% of patients answering ≥80% of ePRO items each week during the course of their treatment. Despite heterogeneous criteria used to define the feasibility of ePRO monitoring, our findings compare favorably with previous publications on the feasibility of digital surveillance in patients with cancer undergoing radiotherapy. An analysis investigating the feasibility of app-based telemonitoring of patients with prostate cancer undergoing treatment defined a feasibility threshold of 70% of patients answering at least 60% of weekly questions over 3 months [22]. In another previous pilot trial, it was reported that 79% of patients with thoracic or pelvic malignancies answered at least 80% of health-related questions during the course of their radiotherapy treatment [23]. Although patients with HNC comprise a heterogeneous group, in our analysis, we could not identify any factor influencing the feasibility of app-based surveillance. Increasing age did not influence acceptance of ePRO monitoring during radiotherapy. A recent Swedish randomized analysis in patients with prostate cancer undergoing radiotherapy found that older age and higher education levels as well as being married correlated with increased daily symptom telemonitoring; in turn, a higher comorbidity burden was associated with decreased questionnaire completion rates [24]. It has been suggested that age may be a critical factor influencing mobile device use, and hence, the feasibility of app-based treatment monitoring; a recent survey found that younger patients may be more open to app-based cancer care [25]. On the other hand, lower proficiency in using mobile devices use did not correlate with item completion rates in a previous trial, suggesting that mobile treatment surveillance may also be feasible for patients with less experience with devices and older age [23,26].

Since patient enrollment in our trial was calculated based on the primary feasibility end point, we cannot dismiss the possibility that, in our data set, the cohort size in the experimental arm was too small to allow the detection of smaller effects regarding patient-related factors that may influence feasibility. Nevertheless, considering the high completion rates, our data suggest that app-based monitoring may provide a feasible approach for monitoring treatment-related symptoms of patients with HNC undergoing radiotherapy.

Due to the aggressive surgical approaches, the high radiotherapy doses, and the large treatment fields used in HNC treatment, there is a considerable risk for higher-grade treatment-related toxicities. Therefore, early detection of therapy-associated toxicities and changes in global and disease-specific quality of life is crucial to inform treating physicians and to allow for early interventions. Additionally, daily app-based ePRO surveillance may assure patients of close medical surveillance during radiotherapy. For patient safety reasons, patients in both the experimental and control arms of our trial received regular weekly physician appointments as per institutional standards and additional appointments, if deemed necessary by either the patient or the health care provider, and no additional medical interventions were scheduled based on the information collected through the app. Nevertheless, patients in the ePRO monitoring arm reported a significantly higher symptom burden upon completion of radiotherapy. As patient characteristics were well balanced between both arms of the trial and higher treatment-related toxicities were unlikely in the experimental arm, it is highly probable that daily symptom monitoring sensitized patients to follow up on their own symptoms more closely and lowered the threshold for reporting those symptoms. This is especially conceivable, as both the global health and most domains of generic quality of life ranked similarly between both study arms. However, as no longitudinal daily or weekly monitoring of disease-specific symptom burden was performed in the standard-of-care group during radiotherapy, no meaningful analysis can be carried out to confirm or rule out a sensitization effect.

In line with the observed high compliance rates for app-based ePRO monitoring, patient satisfaction was high, and several domains of satisfaction were significantly improved by digital monitoring compared to standard-of-care monitoring. Overall, patient satisfaction values, as assessed by the PSQ-18 questionnaire, were comparable to those observed in other trial data sets [27,28]. To the best of our knowledge, no previous trials have investigated patient satisfaction with telemonitoring during radiotherapy based on standardized and validated questionnaires. Our findings suggest that the implementation of close surveillance by itself may provide a perception of regular medical attention and care in patients, for example, by providing a low-threshold means of contacting the treating physicians or nurses in case of worsening or new onset of symptoms. Structured reporting of symptoms may also have prepared patients in the trial group for physical appointments with their health care provider; patients undergoing daily ePRO monitoring rated the time spent with the doctor and interpersonal manners significantly higher in their evaluations. The data underpin the high acceptance of app-based treatment monitoring in patients with HNC. In this respect, ePRO monitoring may also increase treatment compliance. A previous analysis of patients undergoing radiotherapy and concomitant endocrine treatment for breast cancer could demonstrate significant improvements in treatment adherence and completion rates by symptom telemonitoring [29].

### Study Limitations

Despite the prospective randomized setting chosen for our feasibility trial, potential limitations need to be pointed out. Since the trial was primarily designed to assess feasibility, the limited cohort size posed challenges for conducting subgroup analyses and evaluating potential variables that could influence feasibility. As patients were aware of their participation in the experimental and control arms of this trial, it is possible that compliance rates were higher than what might be expected in routine clinical care. To account for this potential limitation, we set a very high feasibility threshold that strongly exceeded that of previous trials [22]. Additionally, patient monitoring was performed on department-owned devices rather than patients’ individual mobile devices. The use of departmental mobile devices was advised due to national and institutional data protection guidelines; it also enabled the enrollment of patients without a mobile device, avoiding a selection bias in favor of app-proficient patients. Therefore, further investigations on patient telemonitoring using patient-owned mobile devices are warranted.

### Conclusions

Taken together, our trial data demonstrate the feasibility of daily app-based treatment surveillance in patients with HNC undergoing radiotherapy. ePRO monitoring in this setting improved reporting of disease- and treatment-related symptom burden and significantly increased patient satisfaction. Further analyses are needed to assess whether our findings hold true outside the context of a clinical trial and if feasibility results are transferrable to other disease sites.

## Appendix

Multimedia Appendix 1 Logistic regression model for the covariates.

Multimedia Appendix 2 CONSORT checklist.

## Abbreviations

APCOT: App-Controlled Treatment Monitoring and Support for Patients With Head and Neck Cancer
EORTC: European Organisation for Research and Treatment of Cancer
ePRO: electronic patient-reported outcome
ES: effect size
HNC: head and neck cancer
H&N35: head and neck cancer module
PRO: patient-reported outcome
PROM: patient-reported outcome measure
PSQ-18: Patient Satisfaction Questionnaire Short Form
QLQ-C30: Quality of Life Questionnaire

## Data Availability

The data sets generated and analyzed during this trial are not publicly available due to the national data protection laws and the order of the University of Freiburg data protection officer of the University of Freiburg Medical Center. Data are available from the corresponding author upon reasonable request.

## References

[1] Sung H, Ferlay Jacques, Siegel Rebecca L, Laversanne Mathieu, Soerjomataram Isabelle, Jemal Ahmedin, Bray Freddie (2021). Global cancer statistics 2020: GLOBOCAN estimates of incidence and mortality worldwide for 36 cancers in 185 countries. CA Cancer J Clin.

[2] Seiwert TY, Salama JK, Vokes EE (2007). The chemoradiation paradigm in head and neck cancer. Nat Clin Pract Oncol.

[3] Nichols A (2022). Randomized trial of radiotherapy versus transoral robotic surgery for oropharyngeal squamous cell carcinoma: long-term results of the ORATOR trial. J Clin Oncol.

[4] Forastiere AA, Goepfert H, Maor M, Pajak TF, Weber R, Morrison W, Glisson B, Trotti A, Ridge JA, Chao C, Peters G, Lee D, Leaf A, Ensley J, Cooper J (2003). Concurrent chemotherapy and radiotherapy for organ preservation in advanced laryngeal cancer. N Engl J Med.

[5] Blanchard P, Landais C, Petit C, Zhang Q, Grégoire V, Tobias J, Burtness B, Ghi M, Janot F, Overgaard J, Wolf G, Lewin F, Hitt R, Corvo R, Budach V, Trotti A, Fortpied C, Hackshaw A, Bourhis J, Pignon J (2016). Meta-analysis of chemotherapy in head and neck cancer (MACH-NC): an update on 100 randomized trials and 19,248 patients, on behalf of MACH-NC group. Annals of Oncology.

[6] Adelstein DJ, Lavertu P, Saxton JP, Secic M, Wood BG, Wanamaker JR, Eliachar I, Strome M, Larto MA (2000). Mature results of a Phase III randomized trial comparing concurrent chemoradiotherapy with radiation therapy alone in patients with Stage III and IV squamous cell carcinoma of the head and neck. Cancer.

[7] Bernier J, Cooper JS, Pajak TF, van Glabbeke M, Bourhis J, Forastiere A, Ozsahin EM, Jacobs JR, Jassem J, Ang K, Lefèbvre J L (2005). Defining risk levels in locally advanced head and neck cancers: a comparative analysis of concurrent postoperative radiation plus chemotherapy trials of the EORTC (#22931) and RTOG (# 9501). Head Neck.

[8] Sprave T, Verma V, Fabian A, Rühle Alexander, Baltas D, Grosu A, Nicolay NH (2022). Cost effectiveness and health-related quality of life of chemoradiotherapy versus radiation therapy alone in elderly head and neck cancer patients. Strahlenther Onkol.

[9] Rühle Alexander, Haehl E, Kalckreuth T, Stoian R, Spohn SKB, Sprave T, Zamboglou C, Gkika E, Knopf A, Grosu A, Nicolay NH (2021). Surviving elderly patients with head-and-neck squamous cell carcinoma-what is the long-term quality of life after curative radiotherapy?. Cancers (Basel).

[10] Daugaard R, Kjaer T, Johansen C, Christiansen J, Andersen E, Nielsen AL, Dalton SO (2017). Association between late effects assessed by physicians and quality of life reported by head-and-neck cancer survivors. Acta Oncologica.

[11] Falchook AD, Green R, Knowles ME, Amdur RJ, Mendenhall W, Hayes DN, Grilley-Olson JE, Weiss J, Reeve BB, Mitchell SA, Basch EM, Chera BS (2016). Comparison of patient- and practitioner-reported toxic effects associated with chemoradiotherapy for head and neck cancer. JAMA Otolaryngol Head Neck Surg.

[12] El Shafie RA, Weber D, Bougatf N, Sprave T, Oetzel D, Huber PE, Debus J, Nicolay NH (2018). Supportive care in radiotherapy based on a mobile app: prospective multicenter survey. JMIR Mhealth Uhealth.

[13] Berkowitz CM, Zullig LL, Koontz BF, Smith SK (2017). Prescribing an app? oncology providers’ views on mobile health apps for cancer care. JCO Clinical Cancer Informatics.

[14] Kongshaug N, Skolbekken J, Faxvaag A, Hofsli E (2021). Cancer patients' perceived value of a smartphone app to enhance the safety of home-based chemotherapy: feasibility study. JMIR Form Res.

[15] Denis F, Lethrosne C, Pourel N, Molinier O, Pointreau Y, Domont J, Bourgeois H, Senellart H, Trémolières P, Lizée T, Bennouna J, Urban T, El Khouri C, Charron A, Septans A, Balavoine M, Landry S, Solal-Céligny P, Letellier C (2017). Randomized Trial Comparing a Web-Mediated Follow-up With Routine Surveillance in Lung Cancer Patients. J Natl Cancer Inst.

[16] Basch E, Deal AM, Kris MG, Scher HI, Hudis CA, Sabbatini P, Rogak L, Bennett AV, Dueck AC, Atkinson TM, Chou JF, Dulko D, Sit L, Barz A, Novotny P, Fruscione M, Sloan JA, Schrag D (2016). Symptom monitoring with patient-reported outcomes during routine cancer treatment: a randomized controlled trial. JCO.

[17] Dai W, Feng W, Zhang Y, Wang XS, Liu Y, Pompili C, Xu W, Xie S, Wang Y, Liao J, Wei X, Xiang R, Hu B, Tian B, Yang X, Wang X, Xiao P, Lai Q, Wang X, Cao B, Wang Q, Liu F, Liu X, Xie T, Yang X, Zhuang X, Wu Z, Che G, Li Q, Shi Q (2022). Patient-reported outcome-based symptom management versus usual care after lung cancer surgery: a multicenter randomized controlled trial. JCO.

[18] Sprave T, Zöller D, Stoian R, Rühle A, Kalckreuth T, Haehl E, Fahrner H, Binder H, Grosu A, Heinemann F, Nicolay NH (2020). App-controlled treatment monitoring and support for head and neck cancer patients (APCOT): protocol for a prospective randomized controlled trial. JMIR Res Protoc.

[19] Singer S, Arraras JI, Chie W, Fisher SE, Galalae R, Hammerlid E, Nicolatou-Galitis O, Schmalz C, Verdonck-de Leeuw I, Gamper E, Keszte J, Hofmeister D (2013). Performance of the EORTC questionnaire for the assessment of quality of life in head and neck cancer patients EORTC QLQ-H&N35: a methodological review. Qual Life Res.

[20] Bjordal K, Hammerlid E, Ahlner-Elmqvist M, de Graeff A, Boysen M, Evensen JF, Biörklund A, de Leeuw JRJ, Fayers PM, Jannert M, Westin T, Kaasa S (1999). Quality of life in head and neck cancer patients: validation of the European Organization for Research and Treatment of Cancer Quality of Life Questionnaire-H&N35. JCO.

[21] Aaronson NK, Ahmedzai S, Bergman B, Bullinger M, Cull A, Duez NJ, Filiberti A, Flechtner H, Fleishman SB, de Haes J C (1993). The European Organization for Research and Treatment of Cancer QLQ-C30: a quality-of-life instrument for use in international clinical trials in oncology. J Natl Cancer Inst.

[22] Tran C, Dicker A, Leiby B, Gressen E, Williams N, Jim H (2020). Utilizing digital health to collect electronic patient-reported outcomes in prostate cancer: single-arm pilot trial. J Med Internet Res.

[23] El Shafie RA, Bougatf N, Sprave T, Weber D, Oetzel D, Machmer T, Huber PE, Debus J, Nicolay NH (2018). Oncologic therapy support via means of a dedicated mobile app (OPTIMISE-1): protocol for a prospective pilot trial. JMIR Res Protoc.

[24] Crafoord M, Fjell M, Sundberg K, Nilsson M, Langius-Eklöf Ann (2020). Engagement in an interactive app for symptom self-management during treatment in patients with breast or prostate cancer: mixed methods study. J Med Internet Res.

[25] Kessel KA, Vogel MM, Kessel C, Bier H, Biedermann T, Friess H, Herschbach P, von Eisenhart-Rothe R, Meyer B, Kiechle M, Keller U, Peschel C, Schmid RM, Combs SE (2017). Mobile health in oncology: a patient survey about app-assisted cancer care. JMIR Mhealth Uhealth.

[26] Haehl E, Rühle A, David H, Kalckreuth T, Sprave T, Stoian R, Becker C, Knopf A, Grosu A, Nicolay NH (2020). Radiotherapy for geriatric head-and-neck cancer patients: what is the value of standard treatment in the elderly?. Radiat Oncol.

[27] Pompili C, Dalmia S, McLennan Battleday F, Rogers Z, Absolom K, Bekker H, Franks K, Brunelli A, Velikova G (2022). Factors influencing patient satisfaction after treatments for early-stage non-small cell lung cancer. J Cancer Res Clin Oncol.

[28] Barber EL, Bensen JT, Snavely AC, Gehrig PA, Doll KM (2016). Who presents satisfied? Non-modifiable factors associated with patient satisfaction among gynecologic oncology clinic patients. Gynecol Oncol.

[29] Yu J, Wu J, Huang O, Chen X, Shen K (2021). A smartphone-based app to improve adjuvant treatment adherence to multidisciplinary decisions in patients with early-stage breast cancer: observational study. J Med Internet Res.

