# Supplementary material for: App-Controlled Treatment Monitoring and Support for Patients With Head and Neck Cancer Undergoing Radiotherapy: Results From a Prospective Randomized Controlled Trial
Source: J Med Internet Res. 2023 Oct 19;25:e46189. doi: 10.2196/46189 (PMC10623226; doi:10.2196/46189)
Supplement: Multimedia Appendix 1 [file jmir_v25i1e46189_app1.docx]

Supplementary

|  |  | Estimate | Std. Error | z value | p-value |
| --- | --- | --- | --- | --- | --- |
| Histology | X.Intercept. | 13.800 | 4.527 | 3.049 | **0.002** |
|  | Non-keratinizing squamous cell carcinoma | 16.532 | 2760.595 | 0.006 | 0.995 |
|  | Keratinizing squamous cell carcinoma | 17.189 | 2568.299 | 0.007 | 0.995 |
|  | Other | -1.172 | 1.542 | -0.760 | 0.447 |
|  | Weeks | -1.433 | 0.497 | -2.882 | **0.004** |
|  | Age | -0.057 | 0.039 | -1.457 | 0.145 |
|  | Gender (Ref: Male) | 1.664 | 0.997 | 1.669 | 0.095 |
| Smoker | Intercept | 14.307 | 4.375 | 3.270 | **0.001** |
|  | 1 | 18.546 | 2528.954 | 0.007 | 0.994 |
|  | Weeks | -1.559 | 0.513 | -3.035 | **0.002** |
|  | Age | -0.061 | 0.038 | -1.617 | 0.106 |
|  | Gender (Ref: Male) | 2.260 | 0.966 | 2.341 | **0.019** |
| Systematic Therapy | Intercept | 12.781 | 4.144 | 3.084 | **0.002** |
|  | 1 | 1.602 | 0.979 | 1.636 | 0.102 |
|  | Weeks | -1.453 | 0.473 | -3.069 | **0.002** |
|  | Age | -0.046 | 0.038 | -1.204 | 0.229 |
|  | Gender (Ref: Male) | 1.564 | 0.866 | 1.806 | 0.071 |
| Treatment | Intercept | 18.691 | 5.318 | 3.515 | **0.000** |
|  | 2 | -1.744 | 1.005 | -1.735 | 0.083 |
|  | 4 | 11.555 | 2269.491 | 0.005 | 0.996 |
|  | Weeks | -1.545 | 0.496 | -3.113 | **0.002** |
|  | Age | -0.104 | 0.046 | -2.276 | **0.023** |
|  | Gender (Ref: Male) | 1.704 | 0.885 | 1.924 | 0.054 |
| Cardiovascular CoMo | Intercept | 14.930 | 4.245 | 3.517 | **0.000** |
|  | 1 | 0.832 | 1.215 | 0.685 | 0.494 |
|  | Weeks | -1.385 | 0.456 | -3.036 | **0.002** |
|  | Age | -0.076 | 0.038 | -1.992 | **0.046** |
|  | Gender (Ref: Male) | 1.344 | 0.885 | 1.519 | 0.129 |
| Neurologic_CoMo | Intercept | 14.618 | 4.215 | 3.468 | **0.001** |
|  | 1 | -0.045 | 1.293 | -0.035 | 0.972 |
|  | Weeks | -1.355 | 0.438 | -3.094 | **0.002** |
|  | Age | -0.073 | 0.039 | -1.863 | 0.062 |
|  | Gender (Ref: Male) | 1.534 | 0.891 | 1.721 | 0.085 |
| Nephrologic_CoMo | Intercept | 14.922 | 4.317 | 3.457 | **0.001** |
|  | 1 | 16.318 | 2116.048 | 0.008 | 0.994 |
|  | Weeks | -1.438 | 0.485 | -2.966 | **0.003** |
|  | Age | -0.071 | 0.038 | -1.882 | 0.060 |
|  | Gender (Ref: Male) | 1.438 | 0.846 | 1.701 | 0.089 |
| Diabetes | Intercept | 14.588 | 4.112 | 3.548 | **0.000** |
|  | 1 | -0.197 | 1.222 | -0.162 | 0.872 |
|  | Weeks | -1.349 | 0.434 | -3.108 | **0.002** |
|  | Age | -0.073 | 0.039 | -1.879 | 0.060 |
|  | Gender (Ref: Male) | 1.564 | 0.863 | 1.811 | 0.070 |
| COPD | Intercept | 15.563 | 4.423 | 3.519 | **0.000** |
|  | 1 | -1.551 | 1.320 | -1.175 | 0.240 |
|  | Weeks | -1.430 | 0.459 | -3.113 | **0.002** |
|  | Age | -0.078 | 0.040 | -1.927 | 0.054 |
|  | Gender (Ref: Male) | 1.491 | 0.869 | 1.716 | 0.086 |
| CCI_diag | Intercept | 29.897 | 4023.337 | 0.007 | 0.994 |
|  | 1 | 0.068 | 5784.623 | 0.000 | 1.000 |
|  | 2 | -15.766 | 4023.334 | -0.004 | 0.997 |
|  | 3 | -16.605 | 4023.334 | -0.004 | 0.997 |
|  | 4 | -15.332 | 4023.334 | -0.004 | 0.997 |
|  | 5 | -16.591 | 4023.334 | -0.004 | 0.997 |
|  | 6 | -16.107 | 4023.334 | -0.004 | 0.997 |
|  | 8 | -16.609 | 4023.334 | -0.004 | 0.997 |
|  | 10 | 0.700 | 7501.052 | 0.000 | 1.000 |
|  | 11 | 1.298 | 6180.136 | 0.000 | 1.000 |
|  | 13 | -1.819 | 10591.120 | 0.000 | 1.000 |
|  | Weeks | -1.350 | 0.451 | -2.997 | **0.003** |
|  | Age | -0.061 | 0.060 | -1.028 | 0.304 |
|  | Gender (Ref: Male) | 1.224 | 1.080 | 1.133 | 0.257 |
